# Epithelial Cell Transformation and Senescence as Indicators of Genome Aging: Current Advances and Unanswered Questions

**DOI:** 10.3390/ijms22147544

**Published:** 2021-07-14

**Authors:** Masatoshi Kitakaze, Ryota Chijimatsu, Andrea Vecchione, Toru Kitagawa, Yuichiro Doki, Hidetoshi Eguchi, Hideshi Ishii

**Affiliations:** 1 Center of Medical Innovation and Translational Research, Department of Medical Data Science, Osaka University Graduate School of Medicine, Suita, Yamadaoka 2-2, Osaka 565-0871, Japan; momokitakaze@gmail.com (M.K.); rchijimatsu@cfs.med.osaka-u.ac.jp (R.C.); toru@kyowakai.com (T.K.); 2 Department of Gastroenterological Surgery, Graduate School of Medicine, Osaka University, Suita 565-0871, Japan; ydoki@gesurg.med.osaka-u.ac.jp (Y.D.); heguchi@gesurg.med.osaka-u.ac.jp (H.E.); 3 Department of Clinical and Molecular Medicine, University of Rome “Sapienza”, Santo Andrea Hospital, via di Grottarossa, 1035-00189 Rome, Italy; andrea.vecchione@uniroma1.it; 4 Kyowa-kai Medical Corporation, Osaka 565-0871, Japan

**Keywords:** genome, aging, cell transformation, senescence, single cell analysis, metabolism

## Abstract

The recent advances in deciphering the human genome allow us to understand and evaluate the mechanisms of human genome age-associated transformations, which are largely unclear. Genome sequencing techniques assure comprehensive mapping of human genetics; however, understanding of gene functional interactions, specifically of time/age-dependent modifications, remain challenging. The age of the genome is defined by the sum of individual (inherited) and acquired genomic traits, based on internal and external factors that impact ontogenesis from the moment of egg fertilization and embryonic development. The biological part of genomic age opens a new perspective for intervention. The discovery of single cell-based mechanisms for genetic change indicates the possibility of influencing aging and associated disease burden, as well as metabolism. Cell populations with transformed genetic background were shown to serve as the origin of common diseases during extended life expectancy (superaging). Consequently, age-related cell transformation leads to cancer and cell degeneration (senescence). This article aims to describe current advances in the genomic mechanisms of senescence and its role in the spatiotemporal spread of epithelial clones and cell evolution.

## 1. Introduction

The chronological age, the number of years since birth, does not necessarily correspond to the same age-linked average physical characteristics reflected in biological functionality of the organs, tissues, and cells [1]. The age-associated physiology is ultimately defined by intrinsic and extrinsic interactions with stress during individual development and growth. Intrinsic aging-related factors and phenotypes are associated with the genetic material and genetic predisposition inherited from parents [2]. Although multiple factors are involved in regulation of physical aging [3], in this article, we have focused on molecular biology of senescence and current advances in the clinical science of aging.

The age of the genome is defined by a composition of the individual genetic traits (genomic information) and acquired traits (internal and external factors). Therefore, the age of the genome can be evaluated using molecular and biological parameters, including specific age-linked characteristics that indicate age-related changes in DNA, RNA, proteins, and/or metabolites in various tissues and cells. These specific markers of aging in epithelial cells will be discussed below.

## 2. Age-Related Genetic Alterations in Normal Tissues

It has been demonstrated that markers of aging are different in each organ [3]. Besides exposure to intrinsic factors, epithelial tissues are constantly exposed to extrinsic factors and stress, resulting in advanced tissue aging. Genome-wide mutations occur in normal epithelium with no morphological abnormalities in the esophagus and accumulate over time, leading to formation of multiple clones [4]. The accumulation of clones is marked by coexistence of transformed/mutated cell populations, derived from the same somatic cells. Smoking and drinking are known to promote the mutation rate, resulting in, for instance, extreme esophageal aging [4]. It can be interpreted as progression of pathological traits due to harmful exposure. It has also been noted in other tissues that external stimuli that damage the genome induce the proliferation of specific clones, thus, advancing tissue aging and facilitating carcinogenesis [5].

In order to understand the survival, shedding, and occupancy of such clones, it is essential to identify the mutated cell population, both at the organ, tissue, and individual cell levels [6]. However, molecular mechanisms of genome aging and specific tissue/cellular targets associated with physical aging remain largely unclear. Suggestively, the age of the genome can be determined by incorporating complex information about single-cell mutations and functional condition of organs and tissues. Such a multilevel approach may provide a treatment solution for various diseases by helping with drug discovery, immunity [7], and design for the prevention of infectious [8] and neurodegenerative diseases [9]. For this purpose, it is necessary to collect basic information at the single-cell/tissue/organ levels about the actual age of genomic content at normal and pathological conditions.

## 3. Systematization of Disease Concept According to Genome Aging Theory

The genome aging science is distinct from previous research findings that elucidated the mechanism of longevity and the complex mechanism of whole organism aging [3,10,11]. However, recent studies on the molecular mechanism of organ and tissue aging, including the whole biological function, such as metabolism, its related diets, and lifestyle-related diseases, i.e., glucose intolerance/insulin resistance, microcirculatory disorder /coagulation system abnormality, and deterioration of cardiac function/cardiac hypertrophy, have indicated important factors that impact the actual age of an individual [12]. Multiomics analysis of the gut microbiota also showed that changes in the host’s glucose gastrointestinal tolerance were associated with aging traits [13]. In these studies, the pathological target could be clarified using various physiological or microbiological methods, showing that a single-cell-based approach is not always necessary.

**Figure 1:**
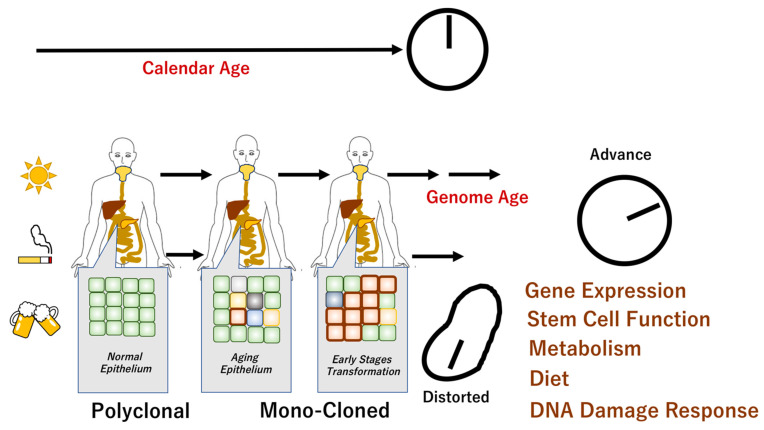
Genome aging and chronological age. It has been shown that there are individuals whose chronological age does not correspond to their biological age. Aging is associated with genomic changes over time, although the specific aging phenotype is influenced by complex internal and external mechanisms. Recent studies revealed that apparently normal epithelium demonstrated polyclonal features. The epithelial monoclonal characteristics underwent cellular transformation that may facilitate carcinogenesis [4,17]. The scheme denotes the mechanisms of chronological age in normal cells and distorted molecular events in cancer cells.

However, to study the age of the genome, we need to register the behavior of individual clones in ordinary somatic cells that do not show morphological abnormalities. Therefore, information at the single-cell level is essential [14]. Single-cell level genomic analysis enables understanding of dynamic cell behaviors, such as individual cell survival, proliferation, cell-to-cell competition, and specialized functions. Cells with internal genome-based deviations are morphologically indistinguishable from normal cells and could not be detected by conventional methods [15]. Furthermore, the definition of genome aging in cancer includes the classification of pathological diagnoses based on cell morphology and identification of the causative genes obtained from the genomic analysis of the lesion [16]. The viewpoint of cancer-related transformations is different from the developmental concept of genome aging. Altogether, the genome aging-related concept relies on comprehensive and systematic understanding of changes in ordinary tissues/cells, which need to be clarified using the latest technological advances in molecular biology (Figure 1).

## 4. Cells as Structural and Functional Units of Living Organisms

Single-cell-based research technologies have a long history of development. Between the 14th and 16th centuries, human morphology and physiology were investigated in the Renaissance era. In the 19th century, Schwann’s cell theory (1839, 1838) was developed, establishing that the cell is “the basic unit of living things” [18]. The theory was supported by numerous discoveries in the latter half of the 19th century, including the discovery of the microscope and establishment of histopathological analysis by Dr. Virchow [19]. Molecular biology, which focuses on investigation of structure and functions of DNA, RNA, and proteins, has been developed in the 1980s and applied to modern medicine. The molecular characteristics were used to define many normal and pathological conditions, including aging. The discovery of super-resolution microscopy in 2014 was marked by the Nobel Prize in Chemistry, indicating that the importance of cell structure and functions continue to be emphasized [20].

To evaluate genome aging according to the cell clone theory, it was suggested to measure, analyze, and model information collected at the single-cell level. To address genome aging in our study, we aimed to use the single-cell-based molecular biology approach. Furthermore, we planned to integrate genomics, population genetics, and cell evolution theory-based methods to collect clone-related information. Alternatively, to the previous studies, we did not aim to address “population genetics”.

Single-cell analysis can be used to detect genomic mutations within individual normal cells that are not morphologically cancerous [21]. It has been indicated that these suggestively “ordinary” cells may contain mutations that are accelerated by aging-based internal and external factors, including smoking and drinking. Future studies should identify which somatic mutations that occur in single-cell units are involved in the phenomena of individual aging and facilitate the development of diseases, including cancer. During the last decade, multiple studies integrated single-cell-based information and evaluated the individual genomic traits using advanced cell analysis technology [15,22,23].

## 5. Measurement of Genome Aging

To investigate genome aging, single-cell transcriptomics and deep tissue proteomics were used in epithelial and stromal cells of various organs, including lungs [24], arteries [25], gastrointestinal tract [26], and skin [27]. Cancer tissue of the defined age was used as the positive control for these investigations. For the negative control group, the epithelium was collected and experimentally induced by iPS technology. Measurements obtained from the normal epithelium of neonatal or childhood diseases were also used as the negative control in studies of genome aging. The negative control tissues were collected during pediatric dermatological surgeries [28].

Although the association with aging of senescent cells and their secretory phenotype (SASP) has been implicated, the molecular mechanisms underlying this phenomenon remains to be understood perfectly. Previous studies elucidated that the exposure to sunlight and ultraviolet light, or genotoxic stimuli, induce numerous damages in genome, and can elicit a DNA damage response, which is considered to be a trigger of SASP [29]. The SASP is associated with the activation of the NF-κB inflammation signaling pathway and thereby enhances the expression of inflammation-related genes, such as inflammatory cytokines. While involved in tissue repair, the SASP is also known to have a cancer-promoting effect by enhancing the inflammatory response; thus, its function is dual [30]. The association of SASP has been proposed in various phenotypes or diseases, such as skin aging [31], asthma [32], inflammatory response in cancer [33], senescent cell clearance [34], autophagy-dependent neural senescence [35], cardiac diseases [36], and muscular dystrophy in mice [37]. As the common mechanism, if not all, the involvement of cancer promoting RAS oncogene, as well as tumor suppressor functions, such as TP53 or p16INK4a, has been proposed [38]. As the senescent phenotypes, telomere shortening and its dysfunction have been associated with the induction of SASP, which could be suppressed by the supplementation of folic acid in the diet of animal models [39]. Nevertheless, it remains to be understood perfectly how we can measure genome aging and whether known SASP factors, such as inflammatory cytokines and DNA damages and responses, could estimate genome aging, as well as whether differences between individuals and organs could be predictive factors of human diseases.

Eventually, cellular reprogramming by iPS factors induced the rejuvenation from differentiated somatic cells to immature undifferentiated states of blast cysts [40]. Although telomere lengths vary among different iPS cell lines, the study of porcine iPS cells indicated that both telomerase-dependent and telomerase-independent mechanisms are involved in telomere reprogramming; insufficient reprogramming may result in the induction of telomere damage and shortening and, furthermore, in chromosomal instability [41], which characterize tumor cells. It has been proposed that further understanding of telomere reprogramming and maintenance may contribute to improvement of the quality of iPS cells [41]. Taken together, further studies will be necessary to understand the issues of telomere length and genome aging. It has been suggested that the telomere shortening, or accumulation of numerous genome damages and responses, may be relatively common physiological processes of living cells, and they may be candidates for the measurement of genome aging.

## 6. Age-Related Clonal Hematopoiesis

Age-related clonal hematopoiesis (ARCH) is defined as the expansion of one or more hematopoietic stem cells or their clones, which harbor specific, disruptive, and recurrent genetic variants [42]. The primary information about ARCH was collected from the study of hematopoietic stem cells. The very first study used hematopoietic stem cells collected from the South African Negro population, using the marker of X-linked enzyme glucose-6-phosphate dehydrogenase (G6PD) [43]. The study demonstrated that the reduced heterozygosity in blood stem cells was increasing with aging [44]. The G6PD allele had been selected due to the functional importance of the enzyme for energy production, although no mutations in this gene were found to support the hypothesis [42]. Previous studies indicated that individuals with ARCH have a 10-fold increased risk for the development of hematological malignancies [45]. Age-related clonal hematopoiesis is more frequent in patients with solid tumors [46].

The prevalence of clonal hematopoiesis among different age groups is highly dependent on the methods used for ARCH detection. The common methods include X inactivation skewing (XIS) (detection of two methylation sites in human androgen receptor genes), single-cell analysis (phylogenetic studies based on epigenetic information), copy number variation (deletions and loss of heterogeneity with increased variant allele frequency (VAF)), and single nucleotide variants (SNV) [42]. Despite current investigations, no therapies for ARCH have been developed, indicating that molecular ARCH targets still remain elusive [42].

Hemizygous selection is supposed to be an alternative characteristic to aging-associated parameters. Hemizygous selection is a phenomenon associated with the competitive advantage of cells that express one parental phenotype, which can be assessed using XIS [47]. Hemizygous selection occurs in differentiating hematopoietic pools during various pathologies, as reported previously. For instance, in women heterozygous for both Wiskott–Aldrich syndrome and G6PD, T cells and platelets showed a single isozyme, whereas red cells and granulocytes expressed both isozymes. This observation suggested that T cells and platelets were not able to differentiate due to masked Wiskott–Aldrich syndrome [48]. Accordingly, the affected individuals had no apparent symptoms [48]. Hemizygous selection with a growth advantage was reported in B cells for carriers of agammaglobulinemia [49]. Furthermore, it was reported that the gene product of X chromosome-linked severe combined immunodeficiency (SCID) syndrome had a direct effect on B and T cells [50].

Hypoxantine guanine phosphoribosyltransferase (HGPRT) was shown to play an important role in folate one carbon (1C) metabolism. The HGPRT-associated deficiency (Lesch–Nyhan disorder) was marked by a skewed distribution of the G6PD phenotype in erythrocytes, granulocytes, and T cells, excluding fibroblasts. This finding suggested that HGPRT is involved in the formation of hematopoietic stem cells, which produces blood cells with a preferential growth advantage [51].

## 7. Age-Related Remodeling of Epithelium

**Figure 2:**
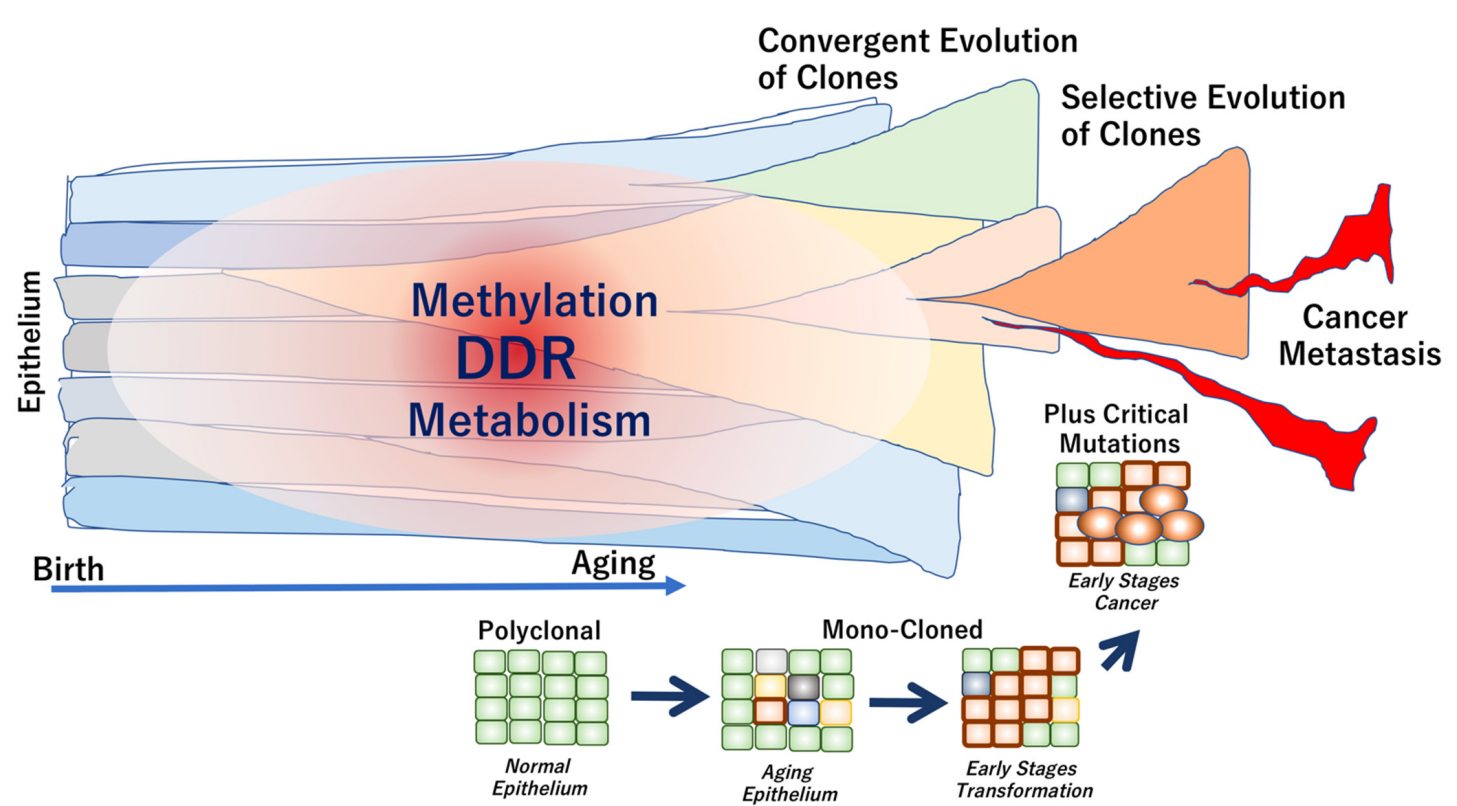
Genome aging associated with epithelial cell transformation and cancer development. In healthy individuals, the normal epithelium is composed of polyclonal components. The clonality is altered toward mono-clonal characteristics during the early stages of transformation, often leading to cancer development. In carcinogenesis, critical driver mutations may occur during exposure to exogenous stimuli or environmental carcinogens. The epithelial clones can be evolved from convergent to selective phenotypes, which are often associated with the development of metastasis and cancer progression [52].

It has been suggested that genome aging is defined by several factors, which are described below. (1) Aging phenotypes slowly evolve from multiple clones due to neutral genome mutations that inevitably occur over the years. (2) During carcinogenesis, strong selective driver gene mutations, such as TP53 mutations, occur independently in each subclone, which coexists with other clones in the tumor tissue. Thus, weak evolutionary neutrality is maintained during cancer dormancy. The length of the dormancy period of time depends on the cancer type. In some early cancers, the dormancy periods are relatively long, indicating a convergent evolution (a phenomenon in which similar traits evolve independently between different cells). (3) Eventually, in advanced cancers with high levels of clinical and biological malignancy, selective pressure is applied via the interaction between clones. Consequently, the adaptive selection results in increased diversity, defined as tumor cell heterogeneity (Figure 2).

## 8. The Mechanisms That Accelerate or Decelerate Genome Aging

### 8.1. DNA Damage and Cellular Senescence

The previous studies indicated that the accumulation of DNA damage is associated with cellular senescence as non-cell-autonomous mechanisms, which play a role in aging-associated diseases. Since the replicative senescence was reported as the phenotype of human fibroblasts, which show a limited proliferative lifespan, with permanent cell cycle arrest from serial passage in culture [53], the previous studies revealed the underlying mechanism. The senescent cells show apparently distinct phenotypes in morphology, being enlarged and flattened, but remain metabolically active in culture [54]. The senescent cells are characterized by the presence of senescence-associated beta-galactosidase (SA-β-gal). The study showed that most somatic cells possess a limited number for the cell proliferation, and the replicative senescence was induced due to the association of telomere shortening. The sensitive assay for measuring telomerase activity demonstrated that telomerase is repressed in normal human somatic tissues but reactivated in cancer, indicating the significance of the control of telomere length in tumor growth [55]. Importantly, the previous studies indicated that the events of telomere shortening are indistinguishable from sites of induced DNA damage in senescent human fibroblasts [56]. The study indicated that the telomeric foci, containing multiple DNA damage response factors, were assembled in senescent cells under the signals of Ataxia Telangiectasia Mutated (ATM) to Tumor Protein P53 (TP53) and p21, which resulted in the induction of G1 phase arrest, although Ataxia Telangiectasia and Rad3-Related Protein (ATR) appears to play a minor role, suggesting that distinct senescence programs can progress in parallel in response to multiple signals [56]. Once telomeres reach a critically short length, it has been shown that the protective structures of telomeres collapse, and several chromosomal ends result in the uncapped structures, which triggers senescence [57]. The mechanism of DNA damage response in telomere-dependent replicative senescent cells elicits other further mechanisms. The previous studies showed that critically shortened telomeres are closely associated with a site of DNA damage and induction of DNA damage in the whole senescent human fibroblasts, which can be detected by nuclear foci of phosphorylated histone H2A.X Variant Histone (H2AX). The H2AX is involved in diseases such as Nijmegen Breakage Syndrome and Ataxia-Telangiectasia. Nijmegen Breakage Syndrome is an autosomal recessive chromosomal instability disorder characterized by early growth retardation, congenital microcephaly, immunodeficiency, and lymphoreticular malignancies [58]. Ataxia-Telangiectasia is characterized by premature aging, i.e., prototype genome instability and multifaceted disorders, such as neurodegeneration, primarily cerebellar atrophy, immunodeficiency, telangiectasia, cancer predisposition, and high sensitivity to DNA damaging agents [59]. Those are associated with ‘aging’ phenomenon. The phosphorylation of H2AX is co-localized with DNA repair and DNA damage checkpoint factors, such as Tumor Protein P53 Binding Protein 1 (53BP1), Mediator Of DNA Damage Checkpoint 1 (MDC1), and NBS1 gene (nibrin, also referred to as p95), as well as the concomitant activation of the DNA damage inducible kinases Checkpoint Kinase 1 (CHK1) and CHK2 [60]. Taken together, the recent study demonstrated that the significance of the mechanism of DNA damage response in telomere-dependent replicative senescent cells is closely relevant to the accelerators of genome aging.

The recent studies demonstrated the significance of the DNA damage response in telomere-independent premature senescent cells. DNA damage accumulates with age. The previous studies indicated that oxidative stress, such as hydrogen peroxide treatment, can cause massive acute DNA double-strand breaks, which are followed by upregulation of TP53 and p21 and cell cycle arrest in the stressed cells, which is similar to senescent cells, suggesting that the mechanism of DNA damage response may be due to an increase in production of reactive oxygen species (ROS) and a decline in DNA repair capacity with age [61]. Interestingly, the previous study indicated that the dysfunctional state of the mechanism of maintaining telomeres, rather than shortening of telomere length, is supposed to be an important factor in inducing a DNA damage response and premature senescence, as shown by the study of mutant of Telomeric Repeat Binding Factor 2 (TRF2) [62]. In the study, dysfunctional, uncapped telomeres were caused by ectopic expression of mutant TRF2 to induce the DNA damage response in mammalian cells, which resulted in the activation of 53BP1, H2AX, Rad17, ATM, and Meiotic Recombination 11 Homolog 1 (Mre11). Taken together, the activation of the cellular senescence mechanism following a DNA damage response was associated with aging, and it could induce the critical signaling pathways of TP53 and Retinoblastoma 1 (RB), the major tumor-suppressor genes, suggesting that the DNA damage response is a common mediator of cellular senescence and, further, that genomic instability plays a causative role in the aging process [63].

Moreover, recent studies elucidated the alterations of signaling organelle, mitochondria, in cellular aging and senescence in endothelial cells [64], ovarian cells [65], fibroblasts [66], neural cells [67], and epithelial cells [68]. Recent studies of several disorders indicated that mitochondrial DNA harbors abnormalities, such as point mutations, deletions, and copy number variations, and that those are involved in mitochondrial dysfunction, suggesting their relevance to aging and senescence [69].

### 8.2. Metabolism

The recent studies exhibited the mechanism through which Nicotinamide adenine dinucleotide (NAD+), a coenzyme for redox reactions of energy metabolism, can influence health and aging biology [70]. The previous studies indicated that NAD+ can directly and indirectly influence many key cellular functions, including aberrant proinflammatory immune cell activation or ‘inflammaging’ [71]; axonal degeneration, which is a precursor to many age-related neuronal disorders and is associated by rapid NAD+ depletion [72]; autophagy, a key cellular catabolic process that involves the enzymatic breakdown of a cell’s cytoplasm or cytoplasmic components, such as damaged or unneeded organelles or proteins, and allows cells to adapt to variable nutrient availability, which is regulated downstream of NAD-Dependent Protein Deacetylase Sirtuin-1 protein (SIRT1), a regulator that causes changes in histone modifications [73]. Taken together, the mechanism can affect metabolic pathways, DNA repair, chromatin remodeling, cellular senescence, and immune cell function, mainly through the involvement of CD38 and Poly(ADP-Ribose) Polymerases (PARPs) and sirtuins in DNA repair, leading to genomic instability [70]. It has been suggested that the therapeutic restoration of NAD+ levels with the NAD+ precursors, nicotinamide riboside and nicotinamide mononucleotide, may be an important approach to treat age-related diseases [70].

The studies over the past several years have shown that sphingolipids and their metabolism plays a role in physiology and diseases in a major cell signaling pathway, and that the lipid–protein interactions can provide useful biomarkers and therapeutic targets [74]. The study of the mechanisms of regulation of sphingolipid catabolism demonstrated that the TP53-dependent DNA damage response regulates the function of neutral sphingomyelinase 2 (nSMase2) at the mRNA level by TP53 in the context of the DNA damage response, which is associated by epigenetic mechanisms, a combination of DNA methylation and histone deacetylation, induced by all-trans retinoic acid (ATRA) [75]. This mechanism is associated by reactive oxygen species (ROS)-dependent function and tumor necrosis factor (TNF) signaling [76]. The study of the sphingolipid species pathway indicated that ceramide is involved in senescence by the increase of total ceramides [74]. It has been suggested that the sphingolipid mechanism is involved at least partially in the induction of the senescence phenotype.

### 8.3. Diet

As expected, it has emerged that reduced food intake and avoiding malnutrition can ameliorate aging and aging-associated diseases in humans, but also in other model organisms [77]. Although the molecular mechanisms that mediate improvement in health and genetic variation during aging have been identified [77], several interventions, including dietary interventions of calorie restriction and intermittent fasting, physical exercise interventions, and genetic interventions of the cell growth signaling pathway [78], have been shown to extend the lifespan in model animals, as well as pharmacological interventions of rapamycin [79] and metformin [80]. Recent studies demonstrated that meal timing is crucial, with both intermittent fasting and adjusted rhythm of feeding improving health and function, and nutritional modulation of the microbiome is also important [77]. Taken together, it has been suggested that interventions that reduce food intake and avoid malnutrition can enhance resistance to DNA damage, genomic instability, and genome aging, and then presumably contribute towards longevity. Recent studies indicated that although amino acids are the building blocks of proteins, they are also signaling molecules and can be used as relatively non-toxic therapeutic approaches to increase or decrease the rate of aging in experimental models [81]. Nevertheless, much more studies undoubtedly will be necessary to understand the signaling pathways activated by amino acids imbalance in aging and aging-related disorders in humans [81].

Recent studies of genome-wide association study have elucidated genes related to increasing of risk of developing metabolic syndrome and related disorders, although the mutation frequency of individual genes was not so high, such that those alterations did not provide enough explanation about which mechanism is involved in the increasing prevalence of metabolic syndromes or related disorders [82]. For example, recent studies in the liver elucidated three transducing nutrient sensing molecules, cAMP responsive element binding protein 3 like 3 (CREB3L3), peroxisome proliferator activated receptor alpha (PPAR), and forkhead box O1 (FOXO1), suggesting that the orchestration of those transcription factor cascades may play a role in the maintenance of metabolic homeostasis.

## 9. Conclusions and Future Perspectives

Genome aging theory helps to elucidate the mechanism of longevity and organism aging. Recent genome aging-related studies addressed glucose intolerance disorder associated with insulin resistance, microcirculatory disorder and coagulation system abnormality, deterioration of cardiac function, and cardiac hypertrophy. The listed pathologies were linked to the actual age of an individual [12]. Furthermore, multiomics analysis of the gut microbiota assessed changes in host’s glucose tolerance in association with aging traits [13]. However, the majority of these studies clarified various physiological or microbiological aspects of genome aging, due to which the single-cell-based approach was mostly ignored. The age of the genome may elucidate, via investigation of clone behavior in ordinary somatic cells that do not show morphological abnormalities, where the collection of information at the single-cell level is essential [83]. Single-cell level genomic analysis may clarify the mechanisms and changes in dynamic cell behaviors during activation of cell survival, proliferation, competition, and functional responses in cells that are morphologically indistinguishable from normal. The single-cell-based approach is the most efficient in situations when small changes could not be detected by conventional methods. Furthermore, genome aging data may be useful in the definition of pathological diagnosis. For instance, cell morphology assessment is currently being used within standard medical care worldwide. The single-cell-based methods may improve morphological identification of early cell transformation and, therefore, increase control over later stages of carcinogenesis. Genomic analysis of the lesion should be able to identify the causative gene and help with design of relevant personalized treatments. In conclusion, technological advances in molecular biology should be used in the assessment of genome aging, which may help to comprehensively and systematically identify small and early changes in ordinary tissues/cells associated with tissue degeneration and carcinogenesis.

## Data Availability

Not applicable on this review article.
